# Use of Genetically Modified Bacteria to Repair Cracks in Concrete

**DOI:** 10.3390/ma12233912

**Published:** 2019-11-26

**Authors:** Zhigang Zhang, Yiwei Weng, Yuanzhao Ding, Shunzhi Qian

**Affiliations:** 1 Key Laboratory of New Technology for Construction of Cities in Mountain Area, Chongqing University, Ministry of Education, Chongqing 400045, China; zhangzg@cqu.edu.cn; 2 School of Civil and Environmental Engineering, Nanyang Technological University, Singapore 637551, Singapore; ywweng@ntu.edu.sg (Y.W.); yuanzhao.ding@ouce.ox.ac.uk (Y.D.); 3 Singapore Centre for 3D Printing, School of Mechanical and Aerospace Engineering, Singapore 639798, Singapore

**Keywords:** concrete, bacterial technique, genetic modification, repairing efficiency

## Abstract

In this paper, we studied the crack-repair by spraying bacteria-based liquid around the cracks in concrete. To enhance the repair efficiency and speed up the repair process, the transposon mutagenesis method was employed to modify the genes of *Bacillus halodurans* and create a mutant bacterial strain with higher efficiency of calcium carbonate productivity by catalyzing the combination of carbonate and calcium ion. The efficiency of crack-repairing in concrete by spraying two kinds of bacterial liquid was evaluated via image analysis, X-ray computed tomography (X-CT) scanning technology and the sorptivity test. The results show that the crack-repair efficiency was enhanced very evidently by spraying genetically modified bacterial-liquid as no microbiologically induced calcite precipitation (MICP) was found within the cracks for concrete samples sprayed using wild type bacterial-liquid. In addition, the crack-repair process was also shortened significantly in the case of genetically modified bacteria.

## 1. Introduction

Concrete has become the second most consumed material after water on earth nowadays, which contributes greatly to modern buildings due to its high strength, durability, low-cost, and availability worldwide in comparison to other construction materials. However, the drawbacks of low tensile strength and inherent brittleness in concrete make it susceptible to the occurrence of cracks. Cracks in concrete are detrimental to the concrete infrastructure since they open a preferable aisle for the ingress of water, harmful ions, and gases. Without immediate and proper repair, initial micro-cracks in concrete are likely to expand in width from the micrometer to millimeter level, which will result in accelerated carbonation of concrete and corrosion of steel reinforcement, eventually endangering the safety of structures and incurring high maintenance costs.

For crack repair, a variety of techniques are available and the repair is mostly conducted manually. The typical repair involves chemical materials (e.g., epoxy systems, polymers, Na_2_CO_3_, Ca(NO_3_)_2_, Ca(HCOO)_2_ et al. [1,2,3,4,5,6]), which is normally expensive, time-consuming, non-compatible with concrete, lacking durability, and hazardous to the environment and health. 

As an alternative strategy to address this crack repairing issue, recent studies on self-healing concrete have drawn lots of attention in the past decade. Under moist environments, concrete has the potential to heal cracks autogenously by the continuous reaction of unhydrated cementitious materials and the precipitation of CaCO_3_, which has been observed in a bridge in Amsterdam in the 1830s [7]. However, such a naturally occurring, self-healing phenomenon is rarely observed since it requires the crack width to be controlled below 100 μm while the crack width in concrete often reaches millimeter sizes even if restrained by a steel rebar [8,9]. 

To address this issue, Engineered Cementitious Composites (ECC), a unique type of fiber-reinforced concrete with tight crack width control, has been used to study the self-healing behavior. It forms multiple micro-cracks typically below 100 μm instead of localized single cracks in the concrete [10,11]. It was found that the micro-crack could heal itself effectively as the transport properties of pre-cracked ECCs recovered to that of virgin ones [12,13]. Meanwhile, the combination of C-S-H and CaCO_3_ were observed within the crack space, and they are believed to come from the further hydration of unhydrated cement particles and supplementary cementitious ingredients, and the precipitation of CaCO_3_ through the wet-dry curing cycle [9,13]. 

Additionally, incorporating bacteria into concrete could also achieve self-healing due to the microbial activity. The bacterial cells inside the concrete is likely to be activated when the air and water get into concrete through cracks. Afterwards the bacteria start producing mineral compounds due to the microbiologically induced calcite precipitation (MICP) process [14,15,16], and subsequently seal the crack naturally. Many researches have been reported to demonstrate that this bio-concrete technology can heal the crack at around 1 mm wide, efficiently.

As aforementioned, research on self-healing concrete has made great strides; nevertheless, existing infrastructures built with conventional concrete cannot heal cracks autogenously. Hence manual repair is still the most practical way to seal the cracks in most cases. However, it is not a trivial task to look for an environmentally friendly and economical repair material that can repair concrete efficiently before it deteriorates from the initial micrometer-sized cracks to millimeter ones. 

In this context, Jonkers et al. developed a kind of liquid containing bacteria and sprayed it on the cracked surface of concrete buildings, which was expected to seal the crack by the MICP process [17]. The bacterial liquid they developed had been applied in a practical project in the Netherlands to repair cracks (200–300 μm wide) on a parking garage floor. This prevented the deterioration of the existing top coating of the floor and minimized the inconvenience to the users of the garage. After six weeks of curing, which is required for the substantial formation of limestone, the effectiveness of this repair work appeared to be noticeable due to greatly reduced water penetration [18]. The successful application of this technique in practice, while very preliminary, does demonstrate the feasibility of employing bacterial liquid to repair micro-cracks for aging concrete infrastructures. 

For the bio-repairing/healing techniques in concrete, urease positive bacteria is normally used due to the production of urease enzyme during the metabolism of bacteria cells. The urease enzyme could catalyze the hydrolysis of urea (CO(NH_2_)_2_) into carbonate (CO_3_^2−^) and ammonia (NH_4_^+^) ions, meanwhile leading to an increase in the pH [19]. On the other hand, the cell wall of the bacteria is negatively charged, which draws Ca^2+^ from the environment, thereby leading to calcite precipitation in cracks due to the reaction of Ca^2+^ and CO_3_^2−^. To make the process more efficient, extra sources of calcium and carbonate, for instance, calcium acetate and urea, are provided [14]. This process increases the complexity of this technique. Moreover, ureolytic bacteria produce ammonia gas during the process of urea hydrolysis, which may cause some health hazards. Furthermore, the repair efficiency of this technique is still relatively low, which requires several ten days to fully repair/seal the micro-cracks, which will hinder its application. 

In this paper, *Bacillus halodurans*, a kind of carbonic anhydrase positive bacteria, is adopted as a repair liquid for crack-sealing in concrete. The carbonic anhydrases from the metabolism of bacteria cells could capture CO_2_ efficiently and convert it to carbonic acid by the catalysis. This characteristic has made carbon capture a much more viable option for energy firms that use fossil fuels [20]. It is also expected to facilitate the generation of calcite by combining the Ca^2+^ (widely exist on the crack surface) and atmospheric CO_2_, which could avoid the typical usage of urea, thus addressing a number of aforementioned issues with its usage. Furthermore, the transposon mutagenesis method is proposed to create a genetically modified bacteria strain aiming to improve the activity of bacteria, subsequently shorten the period of crack-repairing. In a previous study, the directed addition of *Bacillus halodurans* has already shown good compatibility with the cement system, resulting in increased compressive/tensile strength [21]. 

The transposon mutagenesis method has been widely used in other applied microbiology fields (e.g., environmental engineering, electricity generation, bio-production). For example, Ding et al. used the transposon mutagenesis method to create a *Shewanella oneidensis* mutant (CP2-1-S1) leading to a much higher efficiency of heavy metal removal [22]. Yang et al. used the same *Shewanella oneidensis* mutant to improve the electricity generation in the microbial fuel cell (MFC) [23]. Shi et al. also reported that transposon mutagenesis was employed in *Escherichia coli* to achieve high-yield pyruvate production [24]. Lin et al. applied the Tn5 transposon mutagenesis to improve butanol production by *Escherichia coli* [25]. Hemarajata et al. applied the transposon mutagenesis method to the bacteria *Lactobacillus reuteri* and found that the gene *eriC* greatly affects histamine production [26]. Overall, the transposon mutagenesis method has been widely proved as a method to enhance the bacterial process greatly. However, this method has never been used in the healing/repairing of cracks in the concrete to the best of our knowledge.

This study focuses on the use of genetically modified bacteria to repair concrete cracks efficiently. In the following sections, a brief introduction to bacteria cultivation is presented first. Afterward, the characterizations of bacterial activity are given. Finally, assessments of repair efficiency via various methods are reported, including image analysis, X-ray computed tomography (X-CT) scans, sorptivity tests, and scanning electron microscopy (SEM) observation. 

## 2. Bacteria Cultivation and Characterization

### 2.1. Bacterial Strains and Cultures Preparation

In this study, there are two kinds of bacterial strains: *Bacillus halodurans* (wild type) and the mutant one based on a wild type bacterial strain, which is obtained by the transposon mutagenesis method as described in the previous work of Ding et al. [22]. The bacteria was grown and kept in Luria–Bertani (LB; Difco Broth Miller, BD company, Franklin Lakes, NJ, USA) broth (Tryptone 10 g/L, NaCl 10 g/L, Yeast extract 5 g/L;). The bacteria were grown at 37 degrees for 24 h to reach the stationary phase, leading to mature cultures for further usage in spraying. Unless otherwise specified, other bacterial and molecular tools were obtained from the environmental laboratory of Nanyang Technological University. Luria-Bertani broth was adjusted to pH ~9.7 by addition of 1% (w/v, final concentration) Na_2_CO_3_ after sterilizing. In our previous study, it has been reported that the mutant bacterial strain has a relatively higher CaCO_3_ productivity than that of the wild type [21]. 

### 2.2. Characterization of Bacteria via the Growth Curve Measurement

**Figure 1:**
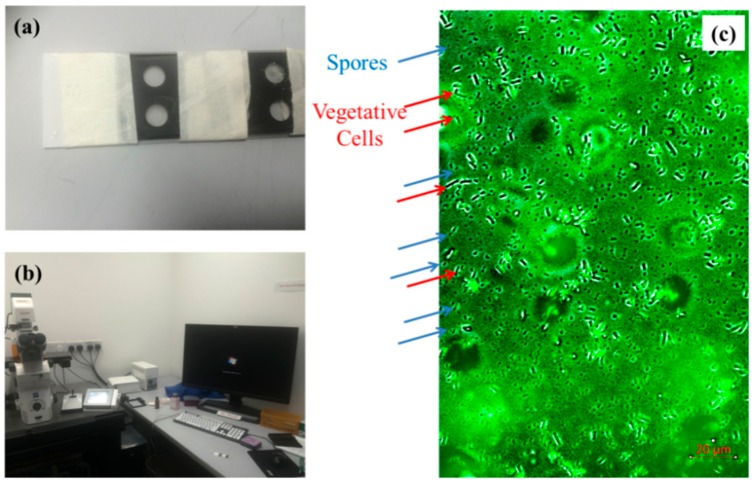
Procedure to calculate the spores and vegetative cells. (**a**) staining process by 4′,6-diamidino-2-phenylindole DAPI; (**b**) Z1-inverted microscopy; (**c**) identification of bacteria and spores based on the microscopy image.

The growth curve was examined by optical density at 600 nm (OD600) under the Tecan M200 microplate reader (Tecan Group Ltd, Mannedorf, Switzerland) or UV-Vis spectrometer. For the Tecan M200 microplate, 198 L of LB medium was mixed with 2 L of wild type (or mutant strain) in each well. Then the OD600 was read by the Tecan M200 microplate every 5 min. For the UV-Vis spectrometer, 19.8 mL of LB medium was mixed with 0.2 mL wild type (or mutant strain) in the 50-mL tubing. In each 2 h, 1 mL culture was taken for examination of the OD600 in the UV-Vis spectrometer. To ensure accurate readings of spores and vegetative cells, the samples were loaded on the glass slide (see Figure 1a) and stained by 4′,6-diamidino-2-phenylindole (DAPI) for 60 min under dark condition. The images were obtained under the ZEISS Axio Observer Z1 Microscope (Baden-Württemberg, Baden-Württemberg, Germany) at 405 nm (see Figure 1b) and processed by IMARIS 7.0 microscopy image analysis software. Under the microscope, the spores and the vegetative cells can be clearly counted (see Figure 1c). 

## 3. Assessment of the Crack-Repairing Efficiency

It’s well known that normal concrete is inherently brittle that makes it difficult to control the crack width at the micron level. In this paper, engineered cementitious composites (ECC), a kind of fiber reinforced concrete with high ductility and excellent crack width control capacity [10], is used to prepare the samples with micro-cracks by pre-loading. The bio-liquid developed in this study will be sprayed on the surface of the cracked samples one time per day to observe the crack-sealing effect. There are two series: series W and M, which means that the surface of samples was sprayed by wild-type bacterial and mutant bacterial bio-liquid, respectively. In the end, the crack repairing efficiency is characterized via digital image processing (DIP) technique, X-ray CT scanning analysis, and sorptivity test.

### 3.1. Specimen Preparation

As shown in Table 1, one ECC mixture was used in the preparation of cracked specimens for repair efficiency observation. The chemical composition and physical properties of fly ash are summarized in Table 2. For this ECC mixture, the crack width after tensile loading could be controlled below 300 μm, which was detailed in reference [27].

For crack image analysis and X-CT scan technique, the uniaxial tensile test was employed on ECC dogbone specimens to produce cracks until failure. The test set-up could be found in reference [21]. After the testing, segments with cracks were cut from the dogbone specimen. The bio-liquid was sprayed on the surface of cracked ECC segments for a number of times (once a day) until the crack was filled by the sealing products. The test was ended after 20 times of spraying even if there was no crack sealing. The specimens were then cut into small pieces for the X-CT scan to observe the distribution of the sealing products within the crack space. Each piece containing one crack had dimensions of 10 mm × 10 mm × 15 mm. 

The sorptivity test was also used to characterize crack-repairing efficiency. The four-point bending test was employed on ECC beam specimens (350 mm in length, 100 mm in width, and 50 mm in height) to induce cracks until failure. Then the segment with cracks was cut from the beam specimen for subsequent bio-liquid spraying. To ensure the same crack pattern on the two series, the segment was then cut again into two halves along the direction that is normal to the crack length. The sorptivity test was conducted for the cut specimens before spraying and after each spraying. Before spraying, the width of each crack is recorded using an optical microscope with a precision of 10 μm.

### 3.2. Image Analysis

**Figure 2:**
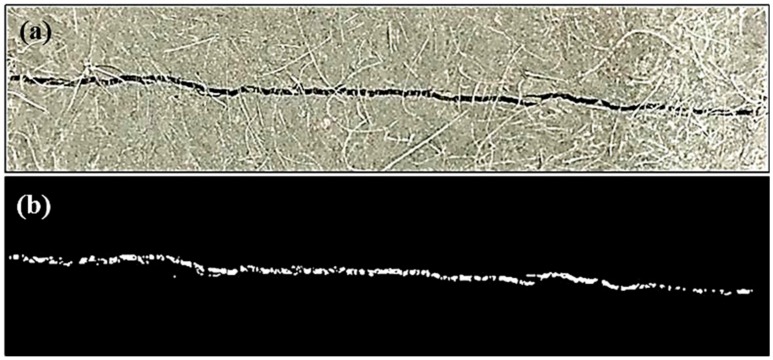
Illustration of crack geometry on the specimen by DIP: (**a**) original image; (**b**) crack revealed in the binary image after processing.

To evaluate the crack-repairing efficiency by bio-liquid spraying, Digital Image Processing (DIP) was employed to identify and analyze crack information, such as crack width, length, and area. All an illustration, one digital photograph captured by a high-definition (HD) camera, was presented in Figure 2, where one crack can be clearly seen.

Cracks in specimens were extracted from these pictures based on a self-compiled MATLAB program. The dimensions of pictures were 3891 pixels by 2917 pixels, whereby each pixel corresponds to 9 μm (resolution of the image). The gray value of the digital photograph was checked against a threshold for the identification of the crack region. A proper gray value must be selected as a threshold to differentiate the crack region from its background so that a binary image can be constructed. The range of gray value was from 0 to 255, and a gray threshold of 80 was selected in order to identify cracks effectively so that pixels with gray values less than 80 were identified as crack regions.

Figure 2a illustrates a raw image directly obtained by a camera device. It can be found that the color of the crack is dark. In this study, a raw, colorful image was transferred into a gray image first; thereafter, pre-processing work was conducted, which included contrast enhancement, removal of the noise points caused by the density mutant, and burr smoothing of the border. In the final step, the Otsu binary method was used to determine the optimal threshold [28], which can effectively distinguish the crack from the surrounding matrix. Consequently, a binary image of crack can be obtained, as shown in Figure 2b. By the use of morphological operation described above, cracks are effectively extracted from the surrounding matrix. Accordingly, geometry indicators, such as crack area, width, and length, are calculated based on the pixel number that the crack contains.

The photograph of the crack was taken by a camera before spraying and after each bio-liquid spraying. The sealing percentage (α) was calculated as Equation (1), where *A_i_* is the initial crack area, *A_t_* is the crack area after spraying.

(1) $$\alpha =\frac{A_{i}-A_{t}}{A_{i}}\times 100\%$$

### 3.3. X-ray CT Scanning

To quantify the amount of precipitation from the MICP process along with the crack depth, the X-ray micro-computed tomography (μ-CT) technique, performed with a SkyScan1173 scanner (Kontich, Antwerp, Belgium), was employed. For this analysis, a representative portion of the sample (mentioned in Section 3.2), including the healed crack, was cut into a small prism measuring 10 mm × 10 mm × 15 mm. The samples were fixed on a micro-positioning stage and scanned using a source voltage and current of 110 kV and 60 μA, respectively. During the scanning, the samples were rotated 360° in steps of 0.2°. A total of 1800 2D projections were obtained for each scan, and a 3D projection was constructed based on these 2D projections.

### 3.4. Sorptivity Test

The sorptivity test was performed in compliance with ASTM C1585 [29]. During this test, due to capillary suction and tight crack width in specimens, water is likely to be sucked up and stored within crack space as soon as the bottom of the cracked specimen is in contact with water. As a result, the mass of the specimen increases over time, which is recorded at a given interval of time (1, 2, 3, 4, 6, 8, 12, 16, 20, 25, 36, 49, 64, 81, 120, and 360 min). 

**Figure 3:**
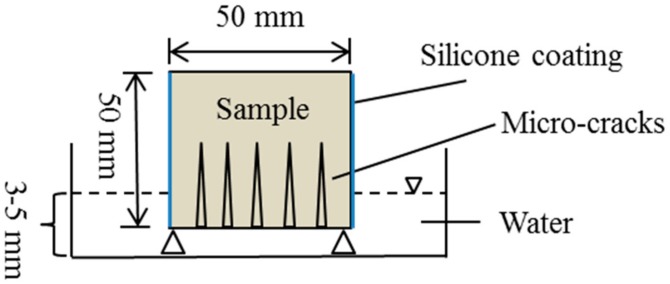
Illustration of the sorptivity test set-up.

Before each test, the specimens were dried in an oven at 60 ± 5 °C for three days. The test set-up was illustrated in Figure 3. As shown in the figure, the immersion depth of the specimen is between 3 and 5 mm. Four vertical faces of the specimen were sealed by a silicone coating to ensure one-directional water-flow through the specimen. Rate of absorption (mm^3^/mm^2^), defined as the variation in mass (g) divided by cross-sectional area of the tested specimen (mm^2^) and density of water at the ambient temperature (g/mm^3^), was plotted against the square-root of time, as detailed in Hall [30]. The sorptivity of the specimen was defined as the slope of the above curve at the initial 6 h of testing. The development of sorptivity with the times of spraying could characterize the repair efficiency. 

## 4. Results and Discussion

### 4.1. The Growth of Bacteria

**Figure 4:**
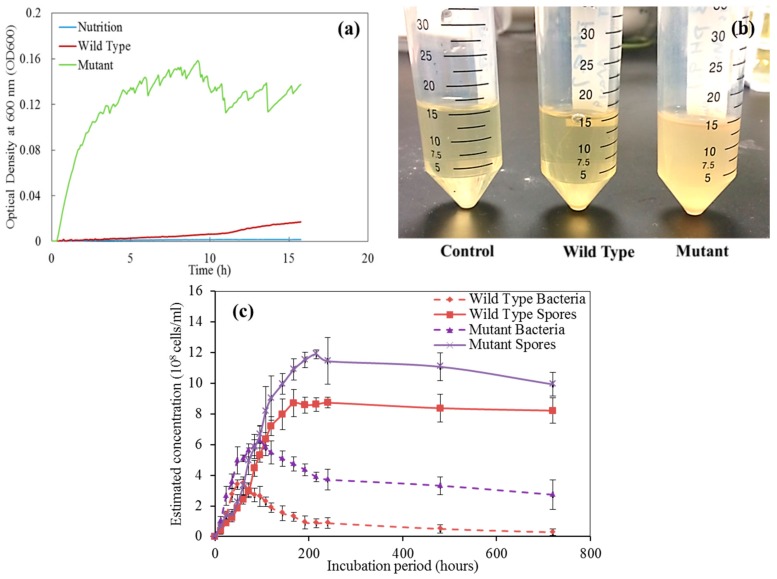
Bacterial growth curves of the control, wild type strain, and mutant strain. (**a**) 16-h growth curve; (**b**) cultures after 16-h growth; (**c**) growth curve over a relatively long time (720 h).

Figure 4 s the growth curves for the two bacteria strains. The mutant strain has a much higher growth rate in the 16-h after inoculation (see Figure 4a). The mutant strain looks milkier, compared to the wild type that is relatively transparent (see Figure 4b). For a relatively long incubation time (720 h), the mutant strain has more vegetative cells and spores, compared to the wild type under the same growth condition (see Figure 4c).

In previous studies, Jonkers et al. grew the *Bacillus subtilis* to perform the healing or repairing of the cracks of the cementitious materials [31,32,33]. This study initially attempted the same bacteria and found it cannot grow well in Singapore. Considering the local environmental conditions, *Bacillus halodurans* was selected for repairing of the cracks in cementitious materials since it is potentially more suitable for growth in the local environment in Singapore with relatively high temperature and humidity. 

The underlying mechanism for bacterial performance improvement is described as follows. This paper, for the first time, improved the bacterial performance in repairing cracks by the transposon mutagenesis method. Previous studies have also suggested that the transposon mutagenesis method might block related genes (*speF* in *Shewanella oneidensis*, *pel psl in Pseudomonas aeruginosa*), further affecting the metabolism pathway (the putrescine and spermidine production) [22,34]. Due to this effect, a relatively stronger/weaker biofilms can be formed (In this paper, mutant bacteria forming stronger biofilms are selected). Many genes (e.g., *psl*, *pel*, *spe*, *bpf*) might be affected during the transposon mutagenesis processing [35,36,37], leading to more robust and cohesive biofilm formation. With more robust and cohesive biofilm, mutant bacteria will have a relatively stable environment [38] to produce more major metabolism products (e.g., CaCO_3_). 

### 4.2. Crack Sealing Estimation by DIP Technique

**Figure 5:**
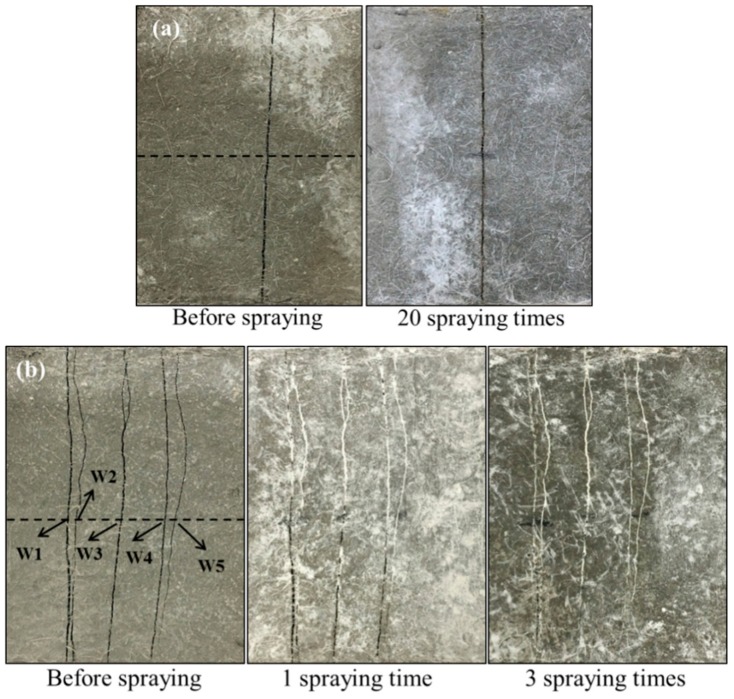
The appearance of cracks before and after spraying: (**a**) wild type bacteria bio-liquid; (**b**) mutant bacteria bio-liquid.

Figure 5 shows the images of cracked samples after repeated bio-liquid spray. The width of cracks along the dashed line was measured by counting pixels using Digital Image Processing (DIP) technique, as described in Section 3.2. As illustrated in Figure 5, for series W, no MICP products were observed on the sample with a crack width of 240 μm even after 20 sprayings (see Figure 5a). On the other hand, a large amount of white residue nearly filled the cracks after three sprayings for series M (see Figure 5b), of which the crack width could be found in Table 2. This phenomenon indicates that, due to the genetic modification, crack-sealing efficiency is enhanced very significantly, which only takes about three days to seal (one spray per day) as compared with previous studies where months are needed [14,15,16,17,18].

**Figure 6:**
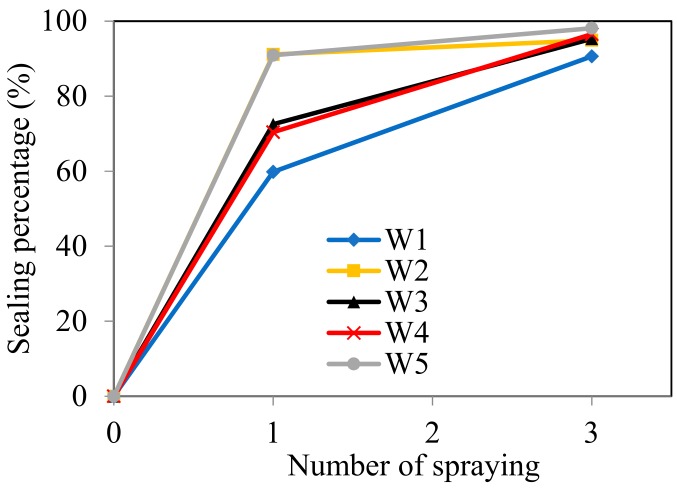
Development of estimated sealing percentage with number of spraying times.

**Figure 7:**
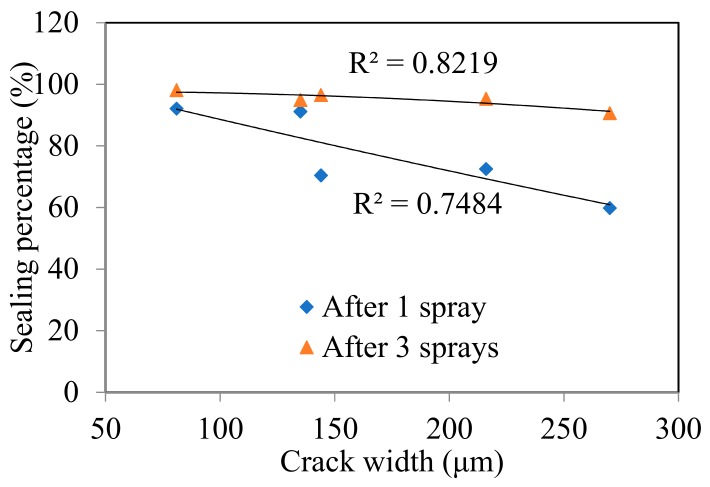
Estimated crack sealing percentage with different crack widths.

Table 3 summarized the measured crack width, crack area before and after spraying, and the calculated sealing percentage by using the DIP technique. Figure 6 illustrated the development of sealing percentage with the number of spraying times. As expected, the crack sealing percentage increases with the number of spraying times. As listed in Table 3, even after only one spraying, the sealing percentage of the crack with 81 μm width reached 92.1%. For the crack with 270 μm width, the sealing percentage could also achieve 59.8%. After three sprayings, all the cracks were sealed almost completely as the sealing percentage of all reached above 90%. Moreover, the crack-sealing percentage presents a liner descending relation with the crack width. As plotted in Figure 7, the sealing percentage decreases as the crack width increases, especially after ones praying. That’s easily understood as a wider crack needs more MICP products to fill. However, after three sprayings, mutant bacteria generated enough MICP products that allowed it fill the crack space almost completely even when the crack width reached 270 μm, which is the largest in this study.

### 4.3. Crack Sealing Phenomenon Observed by X-CT Scanning

**Figure 8:**
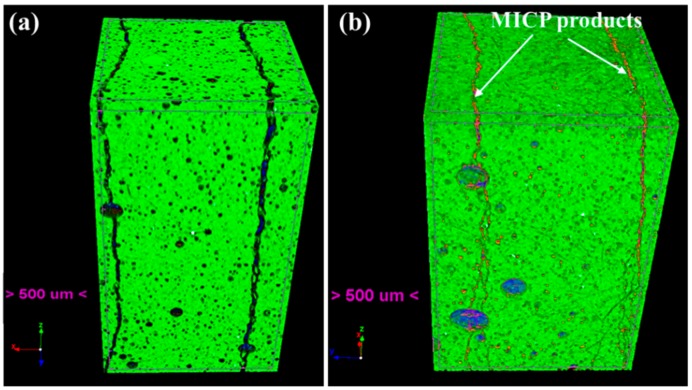
3D view of cracked samples after spraying: (**a**) wild type bacteria and (**b**) mutant type bacteria.

Figure 8 presents the 3D view of samples after bio-liquid spraying, in which the MICP products distributed along the depth of cracks could be observed. It is noted that the top surface of the sample that is shown in Figure 8 is the surface that was in contact with the bio-liquid. Every raw image obtained from the CT scan presented a 2D array of contiguous squares consisting of 256 possible levels (i.e., 0–255 pixels) of gray intensity [39]. The variations in the gray levels referred to different materials and were allocated as: (i) 0–30: Cracks and pores, (ii) 31–100: Healing products, and (iii) 101–255: Cement matrix [39]. As illustrated in Figure 8, in the case M (Figure 8b), MICP products not only appear near the top surface in the crack but also throughout the crack depth. However, there are no MICP products observable within crack space in case W (Figure 8a).

**Figure 9:**
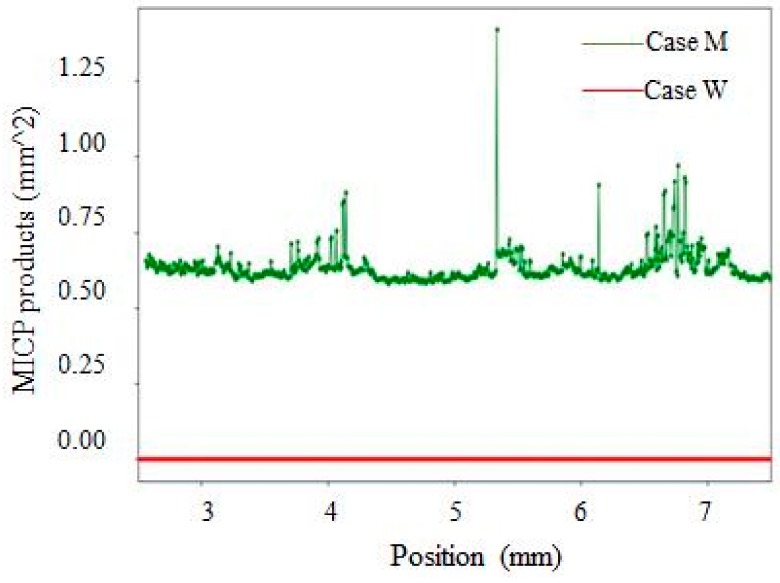
Relationship between the precipitation content and crack depth for both cases.

Figure 9 shows the amount of MICP precipitates as a function of crack depth. It can be seen that in case M, the MICP products distributed not only near the surface of the sample but also deep inside the crack up to 7 mm depth. In contrast, the amount of MICP products at different crack depths is zero for case W. This observation further demonstrates much higher crack repair efficiency when using mutant bacteria strains. 

### 4.4. Sorptivity Test

As reported in Section 4.2, the crack width is very critical for repairing efficiency. The specimen contains six cracks with different widths in this test, which is summarized in Table 4. The crack width of these six cracks ranges from 60 μm to 300 μm with an average value of 128 μm, which has exceeded the threshold for autogenous self-healing in ECC [9]. 

To be consistent with the sample preparation method used for image analysis, the sorptivity test was conducted on pre-cracked samples after three times of spraying for series M (as cracks are fully filled) and 20 times for series W.

**Figure 10:**
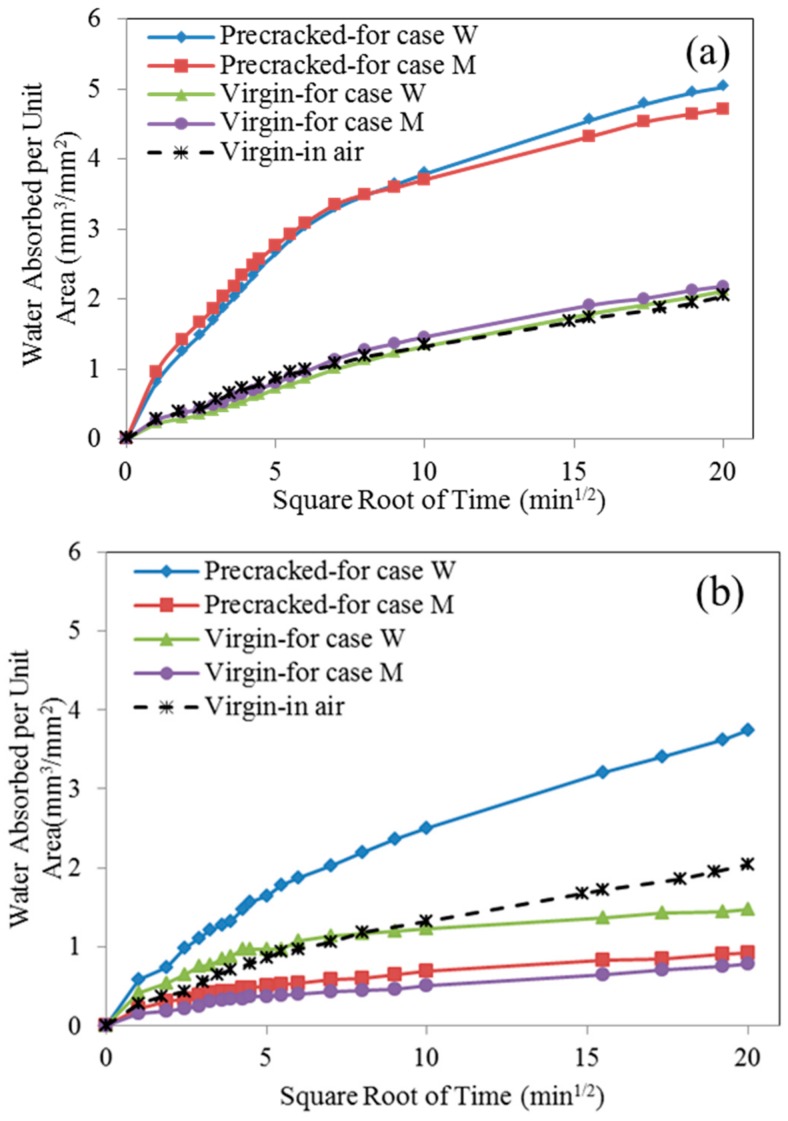
Sorptivity test results for the cracked specimens (**a**) before and (**b**) after repairing.

Figure 10 displays the water absorbed per unit area on samples over time. From Figure 10a, it can be found that, due to the presence of cracks, pre-cracked specimens absorbed much more water as compared with that of virgin ones. That’s because the cracks acted as a capillary pipe to absorb and store water within crack space. For pre-cracked specimens, before repair agent spraying, the water absorptions for both series are nearly indistinguishable (see the blue and red line), which is expected as these two specimens have the same crack pattern (the same specimen was cut in two halves in the direction perpendicular to the cracks). Compared with the virgin samples, the extra water absorbed in pre-cracked samples was mostly contributed by the cracks. Therefore after bio-liquid spraying, the change in water absorption or sorptivity between the pre-cracked and virgin samples can be used to evaluate the sealing efficiency. 

**Figure 11:**
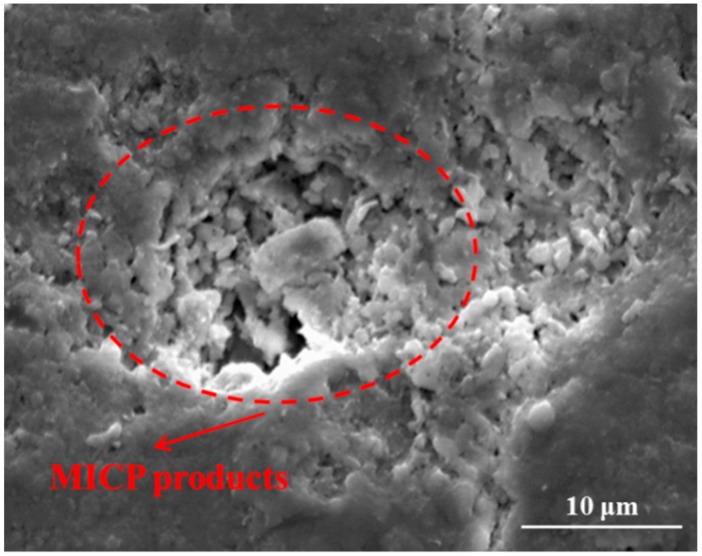
SEM imaging of the surface pore filled with MICP products.

In the comparison of the curves in Figure 10a,b, it can be observed that water absorbed in the pre-cracked specimens decreased after bio-liquid spraying, especially for series M. On the other hand, virgin specimens for both cases also presented less water absorption after bio-liquid spraying. This may be attributed to that the MICP products from bacterial metabolism fill in the pore on the specimen surface for the case M, which is shown in Figure 11. For the case W, no MICP products were found on the specimen surface, reduced water absorption may be explained that the wild type bacteria produce a kind of bio-film after 20 times of spraying, which seals the specimen surface and thereby reduces the absorption of water (will be discussed in details later).

**Figure 12:**
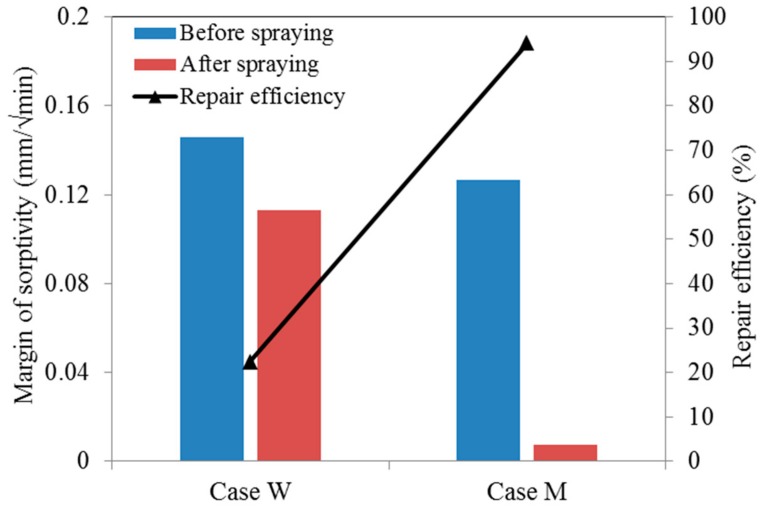
The margin of sorptivity of both cases before and after repair.

Figure 12 displays the margin of sorptivity of pre-cracked specimens, which is defined as the difference in sorptivity of specimens before and after bio-liquid sprayings. It can be seen that, after spraying, the margin of sorptivity of the pre-cracked specimens for series M decreased very distinctly, while only a slight reduction achieved for series W. The decrease percentage for the series W and M, which reflect the repair efficiency, is 22.4% and 94.1%, respectively. This observation again demonstrated that the cracks in concrete could be sealed very effectively by spraying the genetic modified bacteria liquid. 

**Figure 13:**
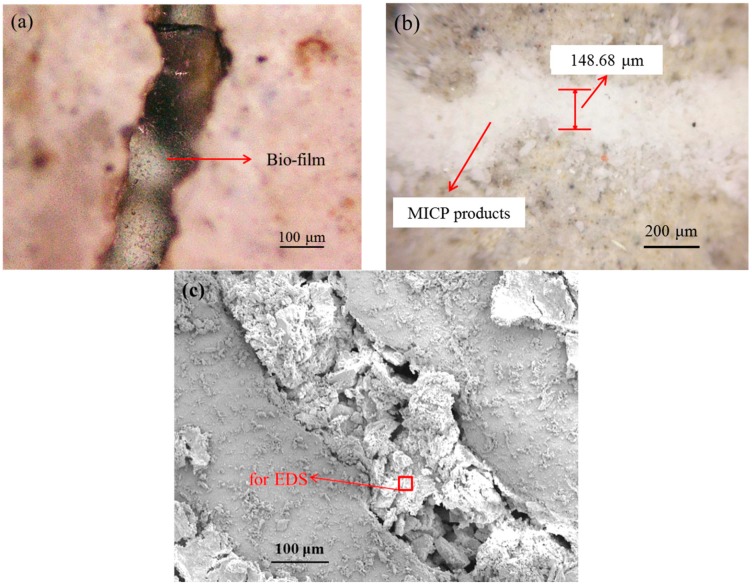
Observations of products within crack space (**a**) bio-film formation after wild type bacteria spraying under an optical microscope; (**b**) MICP products after mutant type bacteria spraying under n aoptical microscope; (**c**) SEM image of sealing products within the crack.

Figure 13 presents the products generated within crack space by bacterial metabolism. Figure 13a shows a kind of bio-film sealed the crack on the specimen of series W under an optical microscope. This is because after spraying wild type bacteria-based bio-liquid for 20 times, the bacterial cells stick to each other and become a polymeric conglomeration of extracellular polysaccharides, proteins, lipids, and DNA [40,41]. This phenomenon also indicates that the wild type bacteria have very low efficiency in catalyzing the combination of carbonate ion and the Ca^2+^ existed within crack space, which is unexpected in the crack repairing technique. However, this kind of film also plays the role of hindering the penetration of water, which explains the decrease in sorptivity of pre-cracked specimens after spraying wild type bacteria. In contrast, for the case M, abundant white crystal-like residues fill the crack space completely, as seen in Figure 13b, which was also observed using a scanning electron microscope (SEM), as shown in Figure 13c. Table 5 list the elemental composition of the products within crack space shown in Figure 13c by Energy dispersive spectroscopy system (EDS), which suggests that the residue products within crack consist of calcium carbonate. It demonstrates that genetically modified bacteria cells could capture CO_2_ efficiently and convert it to carbonic acid by catalysis of carbonic anhydrases, and eventually cause the formation of CaCO_3_ within the crack space by the combination of Ca^2+^ and CO_3_^2−^.

## 5. Conclusions

In this paper, a genetically modified bacterial strain with high activity was proposed to repair the cracks in concrete by spraying, and was expected to enhance the crack-repairing efficiency and speed up the process. The crack-repairing efficiency was evaluated via Digital Image Processing (DIP), X-ray CT scanning technology, and sorptivity test. The specific conclusions are drawn as follows.

(1) The cracks in concrete with width ranging from 80 to 270 μm were sealed near completely by white residue—mutant bacterial metabolism induced products after only three times of spraying. On the other hand, no product was visible in crack even after 20 times of spraying with a wide type of bacterial liquid.
(2) By using X-ray CT scanning technology, a large amount of microbiologically induced calcite precipitation (MICP) products was found to be distributed along with the whole crack depth for the mutant bacterial case, while no products were observed along with the crack depth for wild type bacterial case.
(3) The sorptivity of pre-cracked samples presented a much higher value than that of virgin ones due to the presence of cracks. After spraying mutant bacterial liquid for three times, MICP products, which are postulated to be CaCO_3_ by EDS analysis, were found to distribute within crack space and micro-pore on the surface of samples, and subsequently reduce the sorptivity significantly. For the case of wild type bacteria, a kind of bio-film instead of MICP products was found within a crack, which is plausibly due to its inability to catalyze the combination of Ca^2+^ and CO_3_^2−^ effectively.

Overall, all the results in this study presented that much higher CaCO_3_ productivity of bacterial strain could be achieved by genetic modification, which in turn enhance the crack-repairing efficiency and shorten the repair duration very significantly.

## Figures and Tables

**Table 1:** ECC mixture proportions (by weight).

Cement (C)	Fly Ash (FA/C)	Silica Sand (S/CM)	Water (W/CM)	Water Reducer (WR/C)	PE Fiber (by Volume)
1.0	1.2	0.36	0.25	0.03	0.02

Note: C, FA, S, CM, W, WR, PE are cement, fly ash, silica sand, cementitious material (cement + fly ash), water, water reducer, polyethylene, respectively.

**Table 2:** Chemical composition and physical properties of fly ash (%).

SiO_2_	SiO_2_ + Al_2_O_3_ + Fe_2_O_3_	Na_2_O	CaO	MgO	Fineness	Loss of Ignition	Moisture
50–55	80–85	<1.5	7–9	<5	5.3	<1	<1

**Table 3:** Estimated crack width, area and sealing percentage.

Crack No. (Left-To-Right)	Crack Width (μm)	Crack Area (mm^2^)/Sealing Percentage α (%)		
		Before Spraying	1 Spraying	3 Spraying
W1	270	4.95	1.99/59.8%	0.47/90.6%
W2	135	2.88	0.25/91.1%	0.15/94.9%
W3	216	4.69	1.29/72.5%	0.22/95.3%
W4	144	3.69	1.10/70.3%	0.13/96.5%
W5	81	2.02	0.16/92.1%	0.04/98.1%

**Table 4:** Crack information on the specimen in the sorptivity test.

Crack No.	1	2	3	4	5	6
Crack width (μm)	300	130	100	80	60	100
Average crack width (μm)	128					

**Table 5:** EDS element analysis of products within crack illustrated in Figure 13c.

Element	C	O	Si	Ca
At %	20.75	59.66	1.73	15.28

Notes: Elements with less than 1 atomic percent concentration not shown.
